# Assessing the Reduction of Recurrent Admissions Using OM‐85 for the Treatment of Preschool Wheeze (ARROW): Protocol for a Multi‐Centre, Randomised, Double‐Blind, Placebo‐Controlled Trial

**DOI:** 10.1111/jpc.70434

**Published:** 2026-06-15

**Authors:** Jessica C. Costa‐Pinto, Marisa van Arragon, Katherine J. Lee, Lisa Gold, Peter D. Sly, Cameron C. Grant, Sarah McNab, Peter Vuillermin, Children's Inpatient Research Collaboration of Australia and New Zealand (CIRCAN) Investigator Group, Rebecca Alekzander, Ravichandra Balakrishnamoorthy, Sean Beggs, Habib Bhurawala, Stewart Birt, Katie Bongiolatti, Stephen Brancatisano, Blessy Charles, Roni Cole, Jessica C. Costa‐Pinto, Bailey Craig, Jacqueline Dalby‐Payne, Taya Dowling, Chris Elliot, Daniel Engleman, Wei Qi Fan, Alice Fang, Jane Fata, Catriona Fleming, Jacqueline Fradley, Jeremy Furyk, Arun Gangakhedkar, Lisa Gold, Angus Goodson, Peter Gowdie, Cameron Grant, Kelly Grant, Shehani Gunasekera, Louise Guy, Danielle Hanlon, Rachael Healey, Reena Ho, Sharon Jones, Datta Joshi, Vishal Kapoor, Jonathan Kaufman, Elizabeth Kepreotes, Annie Kilpatrick, Helena Kim, Kristy Kimlin, Christine Kwa, Julia Laing, Anita Lala, Malia Lardelli, Christine Lau, Shirley Lawrence, Katherine J. Lee, Rachel Lee, Jordan Levinter, David Levitt, Hannah Lorking, Lorraine Torrance, April Ly, Ariel Mace, Maricar Santiago Maminta, Payal Mandaliya, Natalie Martin, Brendan McCann, Timothy McDonald, Richard G. McGee, Kathryn McMahon, Sarah McNab, Kristen Mead, Tania Milne, Vasemaca Moi, Nerida Moore, Dylan Mordaunt, Tanya Newnham, Mark Norden, Kirra Philip, Maree Pigdon, Melanie Pillay, Michael Plaister, Jolene Reid, Julia Reid, Nicholas Reid, Kathryn Roberts, Maria Ronan, Thomas Saunders, Jennifer Schaefer, Rachel Schembri, Leonie Shah, Owen Sinclair, Peter Sly, Gail Spence, Jane Standish, Alicia Stanley, Simon Stokes, Scott Sypek, Laurel Teoh, Rachel Teulon, Clare Thomas, David Tickell, Laarni Tundag, Marisa Van Arragon, Peter Vuillermin, Ushma Waida, Matthew Wakeley, Alexandra Wallace, Todd Wallace, Simone Watkins, Ben Watson, Cilla Wyllie‐Schmidt, Rose Ann Yap, Helen Young, Joel Ziffer

**Affiliations:** ^1^ Deakin University Geelong Victoria Australia; ^2^ Barwon Health Geelong Victoria Australia; ^3^ Box Hill Hospital, Eastern Health Melbourne Victoria Australia; ^4^ The University of Auckland Auckland New Zealand; ^5^ Starship Children's Hospital, Te Whatu Ora—Health New Zealand Te Toka Tumai Auckland New Zealand; ^6^ Murdoch Children's Research Institute Parkville Victoria Australia; ^7^ The University of Melbourne Melbourne Victoria Australia; ^8^ Child Health Research Centre The University of Queensland Brisbane Queensland Australia; ^9^ The Royal Children's Hospital Melbourne Victoria Australia

**Keywords:** immunomodulator, OM‐85, Paediatrics, preschool asthma, preschool wheeze, randomised controlled trial

## Abstract

**Aims:**

Acute wheezing illnesses are among the most common reasons preschool‐aged children are admitted to hospital. Readmission rates are high, and novel preventive strategies are required. OM‐85 is an orally administered bacterial lysate that stimulates anti‐viral immune responses and reduces excessive airway inflammation associated with wheezing episodes. The aim of the Assessing the Reduction of Recurrent admissions using OM‐85 for the treatment of preschool Wheeze (ARROW) trial is to determine the efficacy of OM‐85 in preventing hospital readmissions in children admitted to hospital with preschool wheeze. The primary outcome is hospital admission due to an acute wheezing illness during the 12‐month treatment period. Secondary outcomes include the frequency and duration of wheeze episodes and the cost‐effectiveness of the intervention.

**Methods:**

ARROW is a decentralised, Phase 3 randomised, double‐blind, placebo‐controlled trial. We aim to recruit and follow‐up 870 children aged 1 to < 6 years. Participants are randomised to 3.5 mg of OM‐85 or placebo daily for the first 10 days of every month, for 12 months. Health professionals from over 40 hospitals participating in the Children's Inpatient Research Collaboration of Australia and New Zealand (CIRCAN) invite families to the ARROW trial. The ARROW central team organises enrolment, randomisation, medication distribution, and follow‐up, conducting these activities entirely remotely in Australia and both remotely and in‐person in New Zealand.

**Ethics and Dissemination:**

ARROW received ethics approval from The Royal Children's Hospital Human Research Ethics Committee in Australia and the Health and Disability Ethics Committee in New Zealand. Findings will be published in peer‐reviewed international journals.

**Trial registration:** ANZCTR: ACTRN12620001370998 (Registered on 21 December 2020).

## Brief Points

- What is already known on this topic
  ○ OM‐85, an orally administered bacterial lysate, promotes efficient and well calibrated immune responses to viral respiratory tract infections.
  ○ OM‐85 decreases recurrent respiratory tract infections in children and adults.
  ○ Small clinical trials suggest that OM‐85 may prevent wheezing illnesses in children with preschool wheeze; however, larger trials are needed to confirm these findings and assess the impact on hospital readmissions.
- What this paper adds
  ○ ARROW is the first trial of sufficient size to determine whether OM‐85 reduces the risk of hospital readmission among children with preschool wheeze.
  ○ ARROW's decentralised design, with over 40 participating centres, provides a novel and cost‐effective model for conducting large‐scale paediatric clinical trials.
  ○ Our low‐burden methodology for referring participants fosters engagement in clinical trials among inpatient general paediatric teams, which is anticipated to both improve access to clinical trials and support the rapid translation of findings into clinical practice.

## 1 Introduction

Acute wheezing illnesses are a leading cause of hospital admission among preschool‐aged children worldwide [1, 2, 3]. Viral infections trigger 80%–90% of wheeze episodes [4]. International guidelines emphasise the lack of objective evidence of efficacious treatments for preventing preschool wheeze [5]. Available treatments have not been shown to reduce recurrent wheezing episodes in the preschool‐age population and have the potential to cause harm [5, 6, 7, 8, 9]. Accordingly, identifying effective strategies to prevent recurrent preschool wheeze is a key research priority [10], with increasing interest in interventions targeting host responses to viral triggers [11].

OM‐85 (Broncho‐Vaxom) is an oral preparation [12] containing lysates derived from 21 bacterial strains of 8 common bacterial respiratory pathogens, including *Haemophilus influenzae, Streptococcus pneumoniae, Klebsiella pneumoniae, Klebsiella ozaenae, Staphylococcus aureus, Streptococcus pyogenes, Streptococcus viridans* and *Neisseria catarrhalis* [13].

The mechanism of action of OM‐85 centres on the gut‐lung immune axis [14]. OM‐85 binds to pattern‐recognition receptors on intestinal mucosa dendritic cells [14], activating an immune cascade leading to the migration of cells from innate and adaptive branches of the immune system to the lung mucosa, promoting an effective antiviral response [15]. T‐regulatory cells are mobilised to the lungs, correcting the T‐helper cell imbalance and excessive mucosal inflammation and hyperreactivity associated with wheeze episodes [16]. Over 30 years of post‐marketing surveillance indicate a good tolerability profile [17]. Commonly reported adverse events (AEs) are uncommon, mild, and transient, and include gastrointestinal complaints, cough and rash [18].

A 2006 Cochrane review found that, among children with recurrent respiratory tract infections (RTIs), treatment with OM‐85 was associated with a 35.9% relative reduction (95% confidence interval (CI), 22.4% to 49.5%) in acute RTIs compared with placebo [18]. A 2020 meta‐analysis and systematic review concluded that bacterial lysates are a safe and effective add‐on therapy in children with wheeze to reduce exacerbations; however, most studies were performed in school‐aged children [13]. One small, double‐blind, placebo‐controlled, randomised controlled trial (RCT) found that children treated with OM‐85 for 3 months experienced a reduction of 2.18 wheeze episodes over a 12 month period (95% CI, −3.22 to −1.13) compared to placebo, a 38% relative reduction, with weak evidence suggesting a reduction in hospital admissions [19]. Trial limitations included small sample size, group imbalances in baseline characteristics, and exclusion of children treated with inhaled corticosteroids. The need for additional large, high‐quality, double‐blind, placebo‐controlled, RCTs has been identified [20]. More specifically, sufficiently large studies are required to determine whether OM‐85 reduces recurrent hospital admissions due to preschool wheeze.

## 2 Objectives

### 2.1 Primary Objective

- To determine the efficacy of OM‐85 for preventing hospital admissions due to an acute wheezing illness over 12 months in preschool‐aged children with recurrent wheeze and previous wheeze‐related hospital admission.

### 2.2 Secondary Objectives

- To assess the effect of OM‐85 on recurrent wheeze events in preschool‐aged children with reference to: the number of wheeze‐related hospital admissions; number and duration of wheeze episodes; time to readmission; health resource utilisation; cost‐effectiveness; lost productivity; anxiety; and quality of life over 12 months.

### 2.3 Trial Design

ARROW is a Phase 3 multi‐centre RCT of a 12‐month course of OM‐85 or placebo in children aged 1 to < 6 years with recurrent wheeze resulting in at least one hospital admission. Participants are randomised in a 1:1 ratio to OM‐85 or placebo.

## 3 Methods

### 3.1 Trial Setting

ARROW is recruiting participants from over 40 hospitals participating in the Children's Inpatient Research Collaboration of Australia and New Zealand (CIRCAN). Participants are referred to the trial by site doctors and nurses, or by self‐referral. Recruitment, treatment, and data collection are conducted by the ARROW central team, based at Barwon Health, Geelong, Australia, and across seven sites in New Zealand (NZ). Participating units are listed on the trial webpage (https://circan.org/om‐85/).

### 3.2 Eligibility Criteria

Eligibility criteria are shown in Table 1. Hospital admission is defined as a hospital stay of > 6 h from the time of triage to discharge. In 2009, Australia introduced the Four‐Hour National Emergency Access Target requiring 85% of patients to spend < 4 h in the Emergency Department (ED) from the time of arrival [21], and NZ introduced a target of 95% of patients being seen, treated, or discharged from the ED within 6 h [22]. Based on this, the CIRCAN members decided a definition of 6 h would capture all children requiring hospital admission, while minimising misclassification of children who spent significant time waiting due to a low triage category.

**TABLE 1:** Eligibility criteria.

Inclusion criteria
Children aged 1 to < 6 years who in the preceding 12 months have had at least one hospital presentation lasting over 6 h with wheeze **and** at least 2 other episodes of wheeze or whistling in the chest on breathing out, lasting over 6 h, associated with laboured breathing. Parent(s) or legal guardian(s) of the child is/are able to understand the study requirements and willing to provide informed consent.
Exclusion criteria
Known adverse reaction or hypersensitivity to bacterial lysates or to any of the excipients in OM‐85 in accordance with the composition. Known anatomic alterations of the respiratory tract. Chronic respiratory diseases other than preschool wheeze/asthma. These include cystic fibrosis, chronic suppurative lung disease and chronic lung disease of prematurity, defined by a persisting oxygen requirement at a corrected gestational age of 40 weeks. History of malignancy, autoimmune disease, liver, or kidney failure. Treatment with immunosuppressant drugs including methotrexate, cyclosporine, or azathioprine within 30 days prior to enrolment in this trial. Treatment with OM‐85. Limited/no access to an internet connection and/or a phone. Unable to fulfil protocol follow up requirements for > 2 months (e.g., planned overseas travel with limited internet access). Another participant in the same household is concurrently enrolled in the ARROW Trial.

*Note:* For Inclusion criterion 1 hospital admission must have occurred after the age of 12 months. The 2 other episodes of wheeze may have occurred prior to 12 months of age, provided they occurred within the last 12 months.

### 3.3 Recruitment

Potential participants are identified by clinicians at participating sites and are invited to learn more about the ARROW trial and express their interest in participating.

**FIGURE 1:**
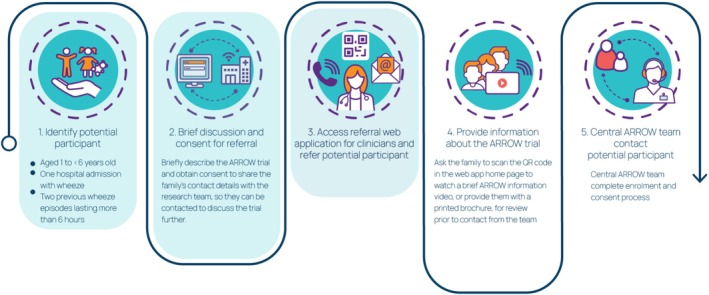
Referral process for clinicians in Australia.

**FIGURE 2:**
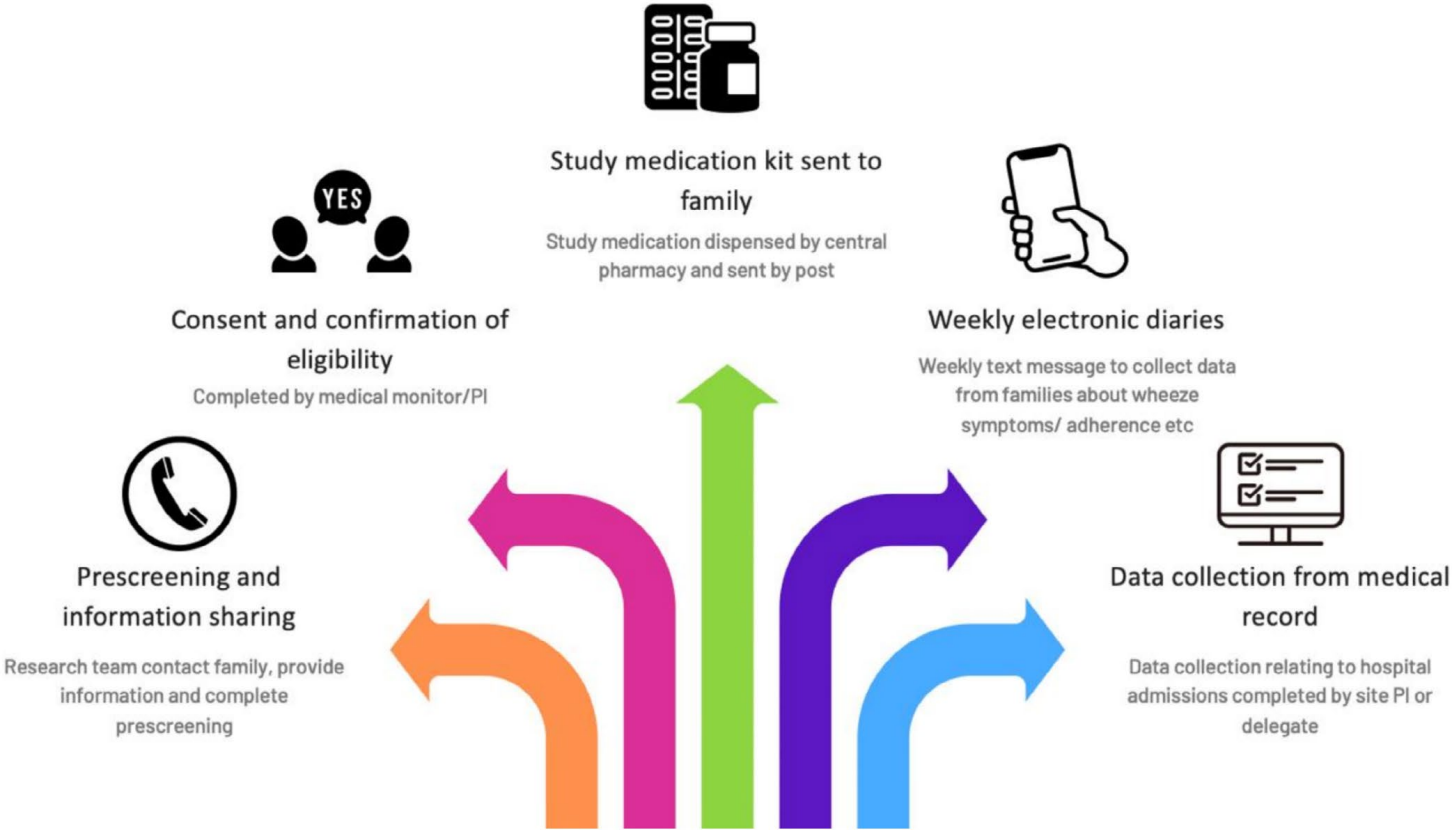
Study procedures in Australia and New Zealand. *Note:* Pre‐screening, information sharing and consent are done remotely in Australia, and either remotely or in person in New Zealand. Consent is completed by doctors in Australia and by nurses in New Zealand. Confirmation of eligibility is always completed by a doctor.

In Australia, doctors use a web application on their devices to refer participants to the ARROW central team (Figure 1), by submitting the family's contact details, minimising the burden on referring clinicians and improving recruitment efficiency. Clinicians provide trial information using a QR code accessed in the web application, linking families to an information video. Once referred, the ARROW team contacts the family by phone to screen eligibility, provide the Participant Information and Consent Form (PICF) and arrange a video conference (Figure 2). Before proceeding, the ARROW team verifies the child's identity and wheeze‐related hospital admission with the relevant hospital site. An ARROW doctor then assesses eligibility, obtains electronic consent from the child's parent or legal guardian, and finalises the enrolment process.

In New Zealand, nurses at each participating site engage with eligible families in person or remotely, providing relevant trial information. The consent and enrolment process is managed and completed by nurses, and a doctor completes the eligibility assessment.

In both countries, the QR code available through the web application, study posters, and information brochures links families to a short study information video and an expression of interest form, allowing for self‐referral. In Australia, families can also access the video and self‐referral form via social media advertisements.

Education and resources are provided to clinicians at participating sites, and engagement is maintained through regular meetings, newsletters, education sessions, and email communications.

### 3.4 Randomisation

Following consent, participants are randomised to either OM‐85 or placebo in a 1:1 ratio, using the Griffith Randomisation System, a web‐based randomisation program [23], in a blinded fashion. The system utilises block randomisation, with variable block sizes, stratified by < 2 versus ≥ 2 admissions for wheeze in the preceding 12 months (including the index admission), age < 3 years or ≥ 3 years, and pharmacy location (Geelong or Auckland). The treatment allocations and block size will be concealed from all participants, trial investigators and research staff until the trial's completion, unless unblinding is required for compelling medical or safety reasons. Pharmacists dispensing the trial medication can unblind participants if required, with all unblinding events recorded in the study database.

### 3.5 Intervention

Participants are provided with capsules containing either 3.5 mg of OM‐85 or a placebo, equal in appearance. The contents of one capsule are administered orally on an empty stomach daily for the first 10 days of each calendar month, for a total of 12 months (120 capsules). Capsules can be opened and their contents poured into a drink, such as milk, water, or fruit juice. Other routine treatments for wheeze can be continued, as determined by the child's treating team, and are not contraindicated during the trial (Table 1). With ongoing parent and community engagement and feedback, we have implemented multiple strategies to maximise participant engagement in the trial and adherence with the study medication and symptom reporting, including text reminders on treatment days and a calendar with stickers to engage participating children. Adherence with the trial medication is defined as the parental report of the participant receiving at least seven doses per month for at least 10 months. Parental report of the number of doses given each month of the study period will be consolidated with the number of remaining capsules reported by parents at the end of the trial.

### 3.6 Data Collection

Data are collected for each participant recruited to the trial as described in Table 2. Participant data are stored using REDCap, a secure web‐based data capture tool [29] hosted by Deakin University, and are only available to research team members as required for their delegated role. Parents or guardians of participating children are sent links to complete the baseline survey, weekly electronic diaries, adherence surveys, and the end‐of‐study survey through REDCap. When a parent/guardian indicates on the electronic diary that their child presented to hospital with a wheeze episode, the ARROW team collects data from the hospital, including admission and discharge time and treatment details. Data are collected for the trial duration, defined as 12 calendar months after commencement of the trial medication. In addition to internal data quality assurance processes, an external monitor completes regular, independent reviews to minimise missing data and ensure data integrity. Incomplete surveys and data collection tools are followed up with participants directly throughout and at the end of the trial, including for participants who have discontinued the study medication for any reason.

**TABLE 2:** Data collected for each participant.

Data collection tool	Timing of completion	Description
Baseline survey	Completed once at start of trial	Demographic information, height, and weight Medical, family, allergy history and social history Completed by parent/guardian
Test for Respiratory and Asthma Control in Kids (TRACK) [24]	At start and end of trial	Validated tool to measure asthma control Completed by parent/guardian
Food Frequency Questionnaire (FFQ) [25]	At start of trial	Information regarding child's diet
Child Behaviour Checklist (CBCL) [26]	At start and end of trial	Validated tool to assess behavioural and emotional problems including anxiety Completed by parent/guardian
Electronic diary	Weekly for duration of trial	Questions about medication changes and symptoms of wheeze If symptoms of wheeze, further questions about health care utilisation, treatment required, details of any hospital admissions, parental time off work, and so forth. Completed by parent/guardian
Adherence survey	Monthly after completion of 10‐day course of trial medication	Number of doses of study medicine administered Side effects of study medication (adverse events) Completed by parent/guardian
Adverse event form	Completed after every reported adverse event (AE) and/or serious adverse event (SAE)^a^	Details of potential AE or SAE, treatment required, outcome, and action required by ARROW team
Hospital survey	Completed after every hospital admission during the trial period	Timing and duration of admission, severity, treatment required and so forth. Data obtained from Principal Investigator at participating hospital, or medical records department if admitted to non‐participating hospital
End of study survey	Completed at end of trial	Child's height and weight Qualitative data regarding family's experience of the trial and suggestions for translating findings into practice
Questionnaires to assess quality of life of parent (EQ5D) [27] and child (PedsQL) [28]	Completed at end of trial	Validated quality of life surveys Completed by parent

^a^ Adverse events are reported by participants through monthly adherence surveys, and by phone or email directly to the ARROW team.

### 3.7 Safety Monitoring

AEs and adherence are reported through weekly surveys or by participants contacting the ARROW team by email or telephone. Assessment of reported AEs is completed by ARROW paediatric doctors and Principal Investigators (PIs), as per the trial Protocol and Safety Monitoring Plan. The PI team may advise that the study medication be withheld or ceased due to concerns about possible AEs, or parents may elect to discontinue for any reason. Participants who discontinue their medication are invited to remain in the trial.

An independent Data and Safety Monitoring Board (DSMB) has been appointed to provide safety oversight and comprises two paediatricians, one respiratory paediatrician, and one biostatistician. The DSMB meets at least annually to review trial safety data and fulfil an advisory role as outlined in the DSMB charter. The trial would only be stopped early based on safety rather than efficacy considerations.

### 3.8 Outcomes

The primary outcome measure is the proportion of participants with a hospital admission (hospital stay lasting > 6 h from the time of triage to discharge) due to an acute wheezing illness over a 12‐month period. Secondary outcomes are shown in Table 3. The 12‐month study period commences on the first day of treatment with the study medication rather than randomisation to allow time for participants to obtain the trial medication and to maintain a consistent treatment and study period for all participants. Commencement of the study medication is deferred where appropriate, for example, in the setting of intercurrent illnesses. If commencement is deferred for more than 3 months, eligibility is reassessed.

**TABLE 3:** Outcome measures.

Primary outcome
Whether the child has a hospital admission due to an acute wheezing illness in the 12‐month period following commencement of the study medication (the study period). A hospital admission is defined as a hospital stay lasting longer than 6 h from the time of triage to the time of discharge.
Secondary outcomes
Time from initiation of the study intervention to subsequent hospital admission due to an acute wheezing illness within the 12‐month study period. Number of hospital admissions (> 6 h from triage to discharge) due to an acute wheezing illness per child over the 12‐month study period. Illnesses resulting in admission are considered discrete if the patient was without symptoms for at least 72 h between the end of one episode and the beginning of the next. Number of wheeze episodes per child over the 12‐month study period. A wheeze episode is defined as a wheezing illness resulting in repeated administration of an inhaled bronchodilator. Once again, illnesses are considered discrete if the patient was without symptoms for at least 72 h between the end of one episode and the beginning of the next. Total cost of health resource utilisation (hospital, community, pharmaceuticals) per child over the 12‐month study period. Parent or guardian loss of productivity (days of work missed/days out of role) over the 12‐month study period. Cost‐effectiveness of the intervention over the 12‐month study period. Mean duration of wheeze episodes (mean per child are used to calculate the group mean) within the 12‐month study period. Quality of life of the child using the PedsQL [28]; and parents using the EQ5D [30] at the end of the 12‐month study period. Anxiety symptoms in child using the Child Behaviour Checklist (CBCL) [26] at the end of the 12‐month study period.

### 3.9 Sample Size

Prior to trial commencement, the most recently published data suggested an annual readmission rate for preschool wheeze of approximately 20% [31]. ARROW was powered to identify a 25% reduction in this rate, which CIRCAN investigators nominated as the minimum effect size required to change current practice. This required 1814 participants (907 per treatment group) based on a two‐sided chi‐squared test with 80% power and an alpha level of 0.05. Allowing for a 20% loss to follow‐up, we planned to recruit 2268 participants (1134 participants per group). However, as of March 2024, the ARROW participants' observed readmission rate for wheezing illnesses over 12 months was 33%, possibly reflecting the recruitment of children with relatively more severe preschool wheeze. This is consistent with a 2023 cohort study reporting a 12‐month readmission rate among children with asthma aged 3–18 years of 34% [32]. Given this, in 2024, we recalculated the required sample size assuming a 35% readmission rate in the placebo group. Retaining a 25% reduction in the admission rate as the minimum effect size needed to change practice (down to 26.25%), we recalculated the required sample size to be 870 participants (435 per treatment group). Allowing for 20% loss to follow‐up, we aimed to recruit 1088 participants (544 per treatment group).

### 3.10 Statistical Methods

Data collection and reporting will follow the CONSORT guidelines, and analyses will be conducted according to the intention‐to‐treat principle, using a treatment policy strategy for all intercurrent events, as detailed in the statistical analysis plan (SAP). The estimand of interest for the primary outcome (hospital admission for wheeze within 12 months) is the odds ratio for OM‐85 compared with placebo, which will be estimated via logistic regression adjusted for stratification factors (age group, number of admissions, and pharmacy location reflecting the country of enrolment). There are no planned interim analyses. Missing data will be managed as per the study protocol and SAP.

The estimand of interest for the binary secondary outcomes is the (adjusted) odds ratio estimated as for the primary outcome. The estimand of interest for the time to readmission is the hazard ratio for the treatment effect, which will be estimated using a Cox Proportional‐Hazards model, censoring participants with no readmissions at the end of the 12‐month follow‐up, adjusted for the stratification factors. The estimand of interest for the number of readmissions and wheeze episodes is the incidence rate ratio, which will be estimated via Poisson regression adjusted for the stratification factors. The estimand of interest for the secondary outcomes of health resource utilisation, parent/guardian loss of productivity, duration of wheeze episodes and parent and child quality of life will be the difference in means between the treatment groups estimated using linear regression adjusted for the stratification factors. All results will be presented with 95% CIs and corresponding *p* values.

The primary economic analysis will be a cost‐effectiveness analysis from a healthcare perspective, comparing the difference in the primary outcome measure with the difference in costs between groups. Costs include the cost of OM‐85 and direct costs of all health service use, including hospital visits, community health services and pharmaceutical use. The secondary economic analysis will assess the impact of the intervention on families including parent‐reported out‐of‐pocket costs, loss of productivity and quality of life outcomes, through a cost‐consequences analysis from a societal perspective that compares the impact of OM‐85 on all measured outcomes to the difference in all costs (to health system, work, and family).

### 3.11 Ethics and Dissemination

Ethics approval was granted by The Royal Children's Hospital Melbourne Human Research Ethics Committee (RCH HREC Reference number: 64007) on 21st September 2021 (Current protocol version 1.7, June 2024) and the Health and Disability Ethics Committee in NZ (Ethics reference: 2022 FULL 11630) on 17th October 2022 (Current protocol version 1.9, May 2024). Approved protocol amendments are communicated to the PI team and to participating sites where relevant. The trial is being conducted in accordance with the ICH Guidelines for Good Clinical Practice [33]. Findings will be published in peer‐reviewed international journals.

### 3.12 Patient and Public Involvement

Before and after commencing the trial, a focus group of parents of preschool‐aged children was held in Australia to seek feedback regarding the information video, promotional materials, PICF, recruitment process, trial requirements and maximising participant engagement. Feedback was considered and incorporated into the study design. During and after their participation in the trial, all participants are invited to provide feedback, and trial processes are adapted as necessary. Frequent feedback is sought from clinicians at participating sites to assist in implementing strategies to maximise engagement and referral numbers.

## 4 Discussion

ARROW will be the first trial of sufficient size to determine whether treatment with a bacterial lysate reduces the risk of hospital readmission among children with preschool wheeze—a primary outcome prioritised by clinicians, policymakers, and families. The concept and protocol were developed in consultation with members of CIRCAN, informed by consumer priorities. Recruitment strategies in Australia and New Zealand differ, but each is well‐suited to its setting. In Australia, the decentralised design is novel and cost‐effective, providing access to families living in regional and remote settings and minimising the burden on referring hospitals. In New Zealand, recruitment aligns with cultural, social, and family preferences, offering both face‐to‐face and remote engagement options for participants.

The ARROW trial has several important strengths. Extensive end‐user engagement from inception, combined with the large number of participating sites, will accelerate the translation of the study's findings into clinical practice [34]. The primary outcome is robust and clinically important. The recruitment strategy, pragmatic design, and minimised participant burden support the feasibility of a trial with sufficient statistical power. The emphasis on providing access to regional and rurally based families in Australia, and on culturally and socially appropriate methods in New Zealand, supports the diversity of participants and, hence, the generalisability of the findings.

Limitations include the reliance on caregivers for the administration of the study medication and data collection. However, primary outcome data are verified by hospital records. The absence of biological sampling prevents the investigation of mechanistic pathways, noting that these are being investigated in other, smaller OM‐85 clinical trials with a higher participant burden (NCT05857930).

ARROW is an efficient, pragmatic trial addressing a critical knowledge gap. If found to be effective, end‐user engagement from inception and throughout the trial, spanning over 40 centres, will facilitate the rapid translation of the findings into practice and policy, leading to improved health outcomes for children with preschool wheeze in Australasia and internationally.

## Author Contributions

J.C.C.‐P. and P.V. drafted the manuscript. All authors participated in study design, and read, contributed to, and approved the final manuscript.

## Funding

This work is supported by NHMRC grants ID#APP1188936 and 2017498, Starship Foundation #SF4218, Cure Kids Grant #3610, Auckland Medical Research Foundation #1122008, Waipapa Taumata Rau—University of Auckland PBRF Fund 2022 and 2023, Te Niwha #TN/SWC/24/UOACG, Lottery Health New Zealand #R‐LHR‐2025‐281951, the HRC Health Delivery Research Grant #24/944/A, and OM Pharma. Funding allocation to parties contributing to the trial is administered by the trial sponsors, Deakin University and Waipapa Taumata Rau—The University of Auckland. Insurance is provided by Deakin University in Australia, and Waipapa Taumata Rau—The University of Auckland in New Zealand. OM‐85 and placebo are supplied by OM Pharma (Broncho‐Vaxom 3.5 mg capsules) free of charge. This is an investigator‐initiated trial, and funding bodies have no control over the trial protocol, conduct of the trial, data analysis, reporting of study findings, publications from the trial in the peer‐reviewed literature, or media releases relating to the trial.

## Disclosure

Participant recruitment began in Australia in March 2022, and in New Zealand in July 2023. As of the 5th May 2026, 1042 participants have been randomised, and recruitment is expected to end in June 2026. These enrolments have resulted from more than 3000 referrals from nearly 500 clinicians across more than 40 sites in two countries.

## Conflicts of Interest

The authors declare no conflicts of interest.

## Data Availability

Data will be stored at Deakin University, Australia. The study protocol, DSMB charter, statistical analysis plan, and data collection tools may be made available on request.

## Appendix A Members of the CIRCAN Investigator Group

Rebecca Alekzander (Kidz First Children's Hospital, Te Whatu Ora—Health New Zealand Counties Manukau, New Zealand)

Ravichandra Balakrishnamoorthy (Flinders Medical Centre, Southern Adelaide Local Health Network, South Australia, Australia)

Sean Beggs (Royal Hobart Hospital, Tasmania, Australia)

Habib Bhurawala (Nepean Hospital, Nepean Blue Mountains Local Health District, New South Wales, Australia)

Stewart Birt (Gosford and Wyong Hospitals, CCLHD, New South Wales, Australia)

Katie Bongiolatti (Royal North Shore Hospital, Northern Sydney Local Health District, New South Wales, Australia)

Stephen Brancatisano (Blacktown and Mount Druitt Hospitals, WSLHD, New South Wales, Australia)

Blessy Charles (The Centenary Hospital for Women and Children, Canberra Health Services, Australian Capital Territory, Australia)

Roni Cole (Sunshine Coast University Hospital, Queensland, Australia)

Jessica C. Costa‐Pinto (Angliss, Box Hill and Maroondah Hospitals, Eastern Health, Victoria, Australia)

Bailey Craig (Women's and Children's Hospital, Adelaide, South Australia, Australia)

Jacqueline Dalby‐Payne (The Children's Hospital at Westmead and Sydney's Children's Hospital Randwick, The Sydney Children's Hospitals Network, New South Wales, Australia)

Taya Dowling (John Hunter's Children's Hospital, Newcastle, New South Wales, Australia)

Chris Elliot (St George Hospital, South Eastern Sydney Local Health District, New South Wales, Australia)

Daniel Engleman (Victorian Virtual Emergency Department, The Northern Hospital, Victoria, Australia)

Wei Qi Fan (Northern Hospital, Epping, Northern Health, Victoria, Australia)

Alice Fang (Box Hill Hospital, Eastern Health, Victoria, Australia)

Jane Fata (Starship Children's Hospital, Te Whatu Ora—Health New Zealand Te Toka Tumai, New Zealand)

Catriona Fleming (Austin Hospital, Melbourne, Victoria, Australia)

Jacqueline Fradley (The Northern Hospital, Epping, Victoria, Australia)

Jeremy Furyk (University Hospital Geelong, Barwon Health, Victoria, Australia)

Arun Gangakhedkar (Waitakere Hospital, Te Whatu Ora—Health New Zealand Waitematā, Auckland, New Zealand)

Lisa Gold (School of Health and Social Development, Deakin University, Victoria, Australia)

Angus Goodson (Tauranga Hospital, Te Whatu Ora—Health New Zealand Hauora a Toi Bay of Plenty, New Zealand)

Peter Gowdie (Casey Hospital, Dandenong Hospital, Monash Children's Hospital, Monash Health, Victoria, Australia)

Cameron Grant (The University of Auckland, Auckland, New Zealand)

Kelly Grant (Royal Hobart Hospital, Tasmania, Australia)

Shehani Gunasekera (Women's and Children's Hospital, Adelaide, South Australia, Australia)

Louise Guy (Waitakere Hospital, Te Whatu Ora—Health New Zealand Waitematā, Auckland, New Zealand)

Danielle Hanlon (Albury Wodonga Health, New South Wales, Australia)

Rachael Healey (Waikato Hospital, Te Whatu Ora—Health New Zealand Waikato, New Zealand)

Reena Ho (Starship Children's Hospital, Te Whatu Ora—Health New Zealand Te Toka Tumai, New Zealand)

Sharon Jones (Deakin University, Victoria, Australia)

Datta Joshi (Casey Hospital, Dandenong Hospital, Monash Children's Hospital, Monash Health, Victoria, Australia)

Vishal Kapoor (Queensland Children's Hospital, Queensland, Australia)

Jonathan Kaufman (Sunshine Hospital, Western Health, Victoria, Australia)

Elizabeth Kepreotes (Broken Hill District Hospital, Far West Local Health District, New South Wales, Australia)

Annie Kilpatrick (Bendigo Health, Victoria, Australia)

Helena Kim (Nepean Hospital, Nepean Blue Mountains Local Health District, New South Wales, Australia)

Kristy Kimlin (Redland Hospital, Queensland, Australia)

Christine Kwa (Casey Hospital, Dandenong Hospital, Monash Children's Hospital, Monash Health, Victoria, Australia)

Julia Laing (Waikato Hospital, Te Whatu Ora—Health New Zealand Waikato, New Zealand)

Anita Lala (Tauranga Hospital, Te Whatu Ora—Health New Zealand Hauora a Toi Bay of Plenty, New Zealand)

Malia Lardelli (Deakin University, Victoria, Australia)

Christine Lau (The Children's Hospital at Westmead, The Sydney Children's Hospitals Network, New South Wales, Australia)

Shirley Lawrence (Kidz First Children's Hospital, Te Whatu Ora—Health New Zealand Counties Manukau, New Zealand)

Katherine J. Lee (The Murdoch Children's Research Institute, Parkville, Victoria, Australia)

Rachel Lee (Kidz First Children's Hospital, Te Whatu Ora—Health New Zealand Counties Manukau, New Zealand)

Jordan Levinter (Deakin University, Victoria, Australia)

David Levitt (Queensland Children's Hospital, Queensland, Australia)

Hannah Lorking (Nepean Hospital, Nepean Blue Mountains Local Health District, New South Wales, Australia)

Lorraine Torrance (Waitakere Hospital, Te Whatu Ora—Health New Zealand Waitematā, Auckland, New Zealand)

April Ly (Starship Children's Hospital, Te Whatu Ora—Health New Zealand Te Toka Tumai, Auckland, New Zealand)

Ariel Mace (Perth Children's Hospital and The Kids Research Institute Australia/University of Western Australia, Perth, Western Australia, Australia)

Maricar Santiago Maminta (Kidz First Children's Hospital, Te Whatu Ora—Health New Zealand Counties Manukau, New Zealand)

Payal Mandaliya (Maitland Hospital, New South Wales, Australia)

Natalie Martin (Christchurch Hospital, Te Whatu Ora—Health New Zealand Waitaha Canterbury, New Zealand)

Brendan McCann (Sunshine Hospital, Western Health, Victoria, Australia)

Timothy McDonald (The Centenary Hospital for Women and Children, Canberra Health Services, Australian Capital Territory, Australia)

Richard G. McGee (Campbelltown Hospital, NSW, Australia and The University of Newcastle, NSW, Australia)

Kathryn McMahon (Werribee Mercy Hospital, Mercy Health, Victoria, Australia)

Sarah McNab(Royal Children's Hospital, Melbourne, Victoria, Australia)

Kristen Mead (Waikato Hospital, Te Whatu Ora—Health New Zealand Waikato, New Zealand)

Tania Milne (The University of Auckland, Auckland, New Zealand)

Vasemaca Moi(Starship Children's Hospital, Te Whatu Ora—Health New Zealand Te Toka Tumai, New Zealand)

Nerida Moore (Royal Darwin Hospital, Northern Territory, Australia)

Dylan Mordaunt (Flinders Medical Centre, Southern Adelaide Local Health Network, South Australia, Australia)

Tanya Newnham (Maroondah Hospital, Eastern Health, Victoria, Australia)

Mark Norden (Albury Wodonga Health, New South Wales, Australia)

Kirra Philip (The Royal Children's Hospital, Victoria, Australia)

Maree Pigdon (Deakin University, Victoria, Australia)

Melanie Pillay (Casey Hospital, Dandenong Hospital, Monash Children's Hospital, Monash Health, Victoria, Australia)

Michael Plaister (The Children's Hospital at Westmead and Sydney's Children's Hospital Randwick, The Sydney Children's Hospitals Network, New South Wales, Australia)

Jolene Reid (Tauranga Hospital, Te Whatu Ora—Health New Zealand Hauora a Toi Bay of Plenty, New Zealand)

Julia Reid (Tauranga Hospital, Te Whatu Ora—Health New Zealand Hauora a Toi Bay of Plenty, New Zealand)

Nicholas Reid (Hutt Hospital, Te Whatu Ora—Health New Zealand—Capital, Coast and Hutt Valley, New Zealand)

Kathryn Roberts (Royal Darwin Hospital, Northern Territory, Australia)

Maria Ronan (Queensland Children's Hospital, Queensland, Australia)

Thomas Saunders (Christchurch Hospital, Te Whatu Ora—Health New Zealand Waitaha Canterbury, New Zealand)

Jennifer Schaefer (The Centenary Hospital for Women and Children, Canberra Health Services, Australian Capital Territory, Australia)

Rachel Schembri (The Murdoch Children's Research Institute, Parkville, Victoria, Australia)

Leonie Shah (Grampians Health, Ballarat, Victoria, Australia)

Owen Sinclair (Waitakere Hospital, Te Whatu Ora—Health New Zealand Waitematā, Auckland, New Zealand)

Peter Sly (Child Health Research Centre, The University of Queensland, Queensland, Australia)

Gail Spence (Kidz First Children's Hospital, Te Whatu Ora—Health New Zealand Counties Manukau, New Zealand)

Jane Standish (University Hospital Geelong, Barwon Health, Victoria, Australia)

Alicia Stanley (Starship Children's Hospital, Te Whatu Ora—Health New Zealand Te Toka Tumai, New Zealand)

Simon Stokes (Frankston Hospital, Peninsula Health, Victoria, Australia)

Scott Sypek (Women's and Children's Hospital, Adelaide, South Australia, Australia)

Laurel Teoh (Te Wao Nui Child Health Service and Hospital, Te Whatu Ora—Health New Zealand—Capital, Coast and Hutt Valley, New Zealand)

Rachel Teulon (Christchurch Hospital, Te Whatu Ora—Health New Zealand Waitaha Canterbury, New Zealand)

Clare Thomas (Sunshine Coast University Hospital, Queensland, Australia)

David Tickell (Ballarat Health Services, Victoria, Australia)

Laarni Tundag (Kidz First Children's Hospital, Te Whatu Ora—Health New Zealand Counties Manukau, New Zealand)

Marisa Van Arragon (Starship Children's Hospital, Te Whatu Ora—Health New Zealand Te Toka Tumai, New Zealand)

Peter Vuillermin (Barwon Health, Geelong, Victoria, Australia)

Ushma Waida (Fiona Stanley Hospital, University of Western Australia Centre for Child Health Research, Perth Children's Hospital, Western Australia, Australia)

Matthew Wakeley (Bundaberg Base Hospital, Queensland, Australia)

Alexandra Wallace (Waikato Hospital, Te Whatu Ora—Health New Zealand Waikato, New Zealand)

Todd Wallace (Deakin University, Victoria, Australia)

Simone Watkins (The University of Auckland, Auckland, New Zealand)

Ben Watson (Royal Children's Hospital, Melbourne, Victoria, Australia)

Cilla Wyllie‐Schmidt(Waikato Hospital, Te Whatu Ora—Health New Zealand Waikato, New Zealand)

Rose Ann Yap (Roan) (Te Wao Nui Child Health Service and Hospital, Te Whatu Ora—Health New Zealand—Capital, Coast and Hutt Valley, New Zealand)

Helen Young (Royal North Shore Hospital, Northern Sydney Local Health District, New South Wales, Australia)

Joel Ziffer (Bendigo Health, Victoria, Australia)
